# Biomarkers for Personalised Primary or Secondary Prevention in Cardiovascular Diseases: A Rapid Scoping Review

**DOI:** 10.3390/ijms26199346

**Published:** 2025-09-24

**Authors:** Chantal Babb de Villiers, Elena Plans-Beriso, Chaitanya Erady, Laura Blackburn, Hayley Wilson, Heather Turner, Isla Kuhn, Cristina Barahona-López, Paul Diez-Echave, Orlando Romulo Hernández, Nerea Fernández de Larrea-Baz, Dafina Petrova, Ramon Cierco Jimenez, Pablo Fernández-Navarro, Esther García-Esquinas, Fernando Rodríguez-Artalejo, María José Sánchez, Víctor Moreno, Marina Pollán, Beatriz Perez-Gomez, Mark Kroese

**Affiliations:** 1 PHG Foundation, University of Cambridge, Cambridge CB2 8RN, UKlaura.blackburn@phgfoundation.org (L.B.); hayley.wilson@phgfoundation.org (H.W.);; 2 CIBER of Epidemiology and Public Health (CIBERESP), 28029 Madrid, Spainv.moreno@iconcologia.net (V.M.);; 3 Department of Epidemiology of Chronic Diseases, National Centre for Epidemiology, Instituto de Salud Carlos III, 28029 Madrid, Spain; 4 Cambridge University Medical Library, Cambridge CB2 0SP, UK; 5 Instituto de Investigación Biosanitaria ibs.GRANADA, 18012 Granada, Spain; 6 Escuela Andaluza de Salud Pública (EASP), 18011 Granada, Spain; 7 Medical Oncology, Hospital Universitario Virgen de las Nieves, 18014 Granada, Spain; 8 International Agency for Research on Cancer, World Health Organisation, Evidence Synthesis and Classification Branch, 69366 CEDEX 07 Lyon, France; ciercor@iarc.who.int; 9 Department of Preventive Medicine and Public Health, Universidad Autónoma de Madrid, 28029 Madrid, Spain; 10 IMDEA-Food Institute, CEI UAM+CSIC, 28049 Madrid, Spain; 11 Oncology Data Analytics Program, Catalan Institute of Oncology (ICO), L’Hospitalet de Llobregat, 08908 Barcelona, Spain; 12 Colorectal Cancer Group, ONCOBELL Program, Institut de Recerca Biomedica de Bellvitge (IDIBELL), L’Hospitalet de Llobregat, 08908 Barcelona, Spain; 13 Department of Clinical Sciences, Faculty of Medicine and Health Sciences, Universitat de Barcelona, 08036 Barcelona, Spain; 14 Institute of Complex Systems, University of Barcelona, L’Hospitalet de Llobregat, 08907 Barcelona, Spain

**Keywords:** precision medicine, biomarker, cardiovascular disease, prevention, early detection

## Abstract

Cardiovascular diseases (CVDs) are the leading cause of morbidity and mortality globally. Early detection and personalised prevention strategies are crucial for reducing the burden of CVD. The use of biomarkers plays a pivotal role in identifying individuals at risk and facilitating timely interventions. This rapid scoping review aims to identify and evaluate current research on biomarkers used for primary and secondary personalised prevention of CVD, highlighting evidence gaps and the integration of digital technologies. A comprehensive search was conducted in Medline and Embase databases from January 2020 to February 2023. Joanna Briggs Institute (JBI) Manual for Evidence Synthesis and PRISMA-ScR guidelines were followed. A total of 775 studies were included, with ischemic heart disease (IHD) and stroke being the most investigated CVDs. Molecular, cellular, imaging, physiological, and anthropometric biomarkers were included. Molecular biomarkers, particularly genetic and biochemical, were the most researched. For secondary prevention, there was considerable research using imaging biomarkers. Genetic biomarker research was the most frequent category of biomarker identified, particularly using variant analysis and polygenic scores, followed by biochemical, imaging, and physiological biomarkers. There was also evidence of the integration of artificial intelligence to enhance the predictive capabilities of these biomarkers. Despite progress, research gaps were identified for less common CVDs, such as aortic aneurysm and nonrheumatic valvular heart disease, and limited research investigating other molecular biomarker types, such as epigenetics and transcriptomics.

## 1. Introduction

Cardiovascular diseases (CVDs) are a leading cause of morbidity and mortality worldwide. The development and progression of CVD are influenced by a complex interplay of genetic predispositions, environmental exposures, and lifestyle choices, with significant differences across demographic factors including age, sex, and gender. It is estimated that 80% of cardiac events and strokes are preventable, and 25% to 75% may be prevented through intervention and early detection [1]. Accurate and cost-effective ways for cardiovascular risk discrimination would allow the deployment of more effective prevention treatment strategies, but appropriate tools for detection have been lacking [2]. Identification of appropriate biomarkers to enhance risk prediction and reduce disease prevalence by facilitating earlier interventions should result in better health outcomes.

In recent years, innovative health research has moved rapidly towards a new paradigm of analysing, processing, and combining unprecedented sources and quantities of data of various kinds, e.g., environmental, clinical, socio-demographic, epidemiological, and biological. This has created new opportunities to better understand the origin and evolution of chronic diseases and facilitates the design of new targeted strategies to prevent, detect, or treat at an individual level, which is known as ‘personalised medicine’.

According to the European Council Conclusion on personalised medicine for patients (2015/C421/03), the term ‘personalised medicine’ defines a medical model that characterises the genotypes, phenotypes, lifestyle, and environmental exposures of individuals. The goal is to tailor the right therapeutic strategy for the right person at the right time and/or to determine disease predisposition and/or to provide timely and targeted prevention [3]. Personalised prevention, a subset of personalised medicine, is an important factor in terms of public health. Personalised prevention aims to prevent the onset, progression, and recurrence of disease by adopting targeted interventions, considering biological information, environmental and behavioural characteristics, and the socio-economic and cultural context of individuals [4]. These interventions should be timely, effective, and equitable to maintain the best possible balance in the lifetime health trajectory.

In this context, non-communicable chronic diseases (NCDs) need specific attention due to their rising incidence and mortality and their impact in terms of disability-adjusted life years [5,6,7]. According to the World Health Organisation [8], each year, NCDs kill 41 million people, equivalent to 74% of all deaths globally. Within the EU, NCDs are estimated to account for 80% of the overall burden of disease, and, in 2021, one third of EU adults were reported to be suffering from a chronic condition [9]. According to the World Health Organisation (WHO), in 2019, circulatory diseases accounted for over 17.9 million deaths or 32% of all global deaths [10]. There is already a significant body of knowledge about primary and secondary prevention strategies for CVD and its associated risk factors [7,11]. Given the above, in 2019, the PRECeDI (Personalised medicine for disease prevention) consortium recommended the identification of biomarkers for the prevention of chronic diseases to support stratification of populations by indicating individuals’ risk or resistance to disease and their possible response to drugs, guiding preventive interventions [12]. Current research focuses on the identification of biomarkers that will aid in the more precise identification of which individuals can benefit from specific preventive strategies or to design and adapt therapies for specific patients or groups of patients [13]. The question is whether there is any biomarker or combination of biomarkers that can improve the identification of subgroups of individuals with different risks of developing a particular disease. Specific preventive strategies and interventions for each subgroup could have an impact on their clinical outcomes.

The aims of this scoping review are to showcase current research on biomarkers that can help improve primary or secondary CVD prevention strategies by better identifying subgroups of individuals at different risk(s); highlight evidence gaps in research on the use of biomarkers in personalised prevention; and examine their integration with digital technologies, such as the use of wearable devices for measuring physical and physiological functions and use of artificial intelligence (AI).

This research is part of the ‘PeRsOnalised Prevention roadmap for the future HEalThcare’ (PROPHET) project, funded by the European Union’s Horizon Europe research and innovation programme and UK Research and Innovation. It is linked to the International Consortium for Personalised Medicine (https://www.icpermed.eu/ (accessed on 7 April 2025)). The PROPHET project seeks to describe the current research and identify the gaps in personalised preventive approaches in order to inform the development of a Strategic Research and Innovation Agenda (SRIA) for the European Union.

## 2. Methods

A scoping review methodology was developed as part of a larger project that included three parallel reviews (for cardiovascular diseases, cancer, and neurodegenerative diseases); this paper presents the findings for the cardiovascular disease review [14]. We conducted an overview of the research landscape and identified gaps in the research literature on biomarkers for personalised primary and secondary prevention of CVD.

### 2.1. Protocol

The review process followed the Joanna Briggs Institute (JBI) Manual for Evidence Synthesis and Preferred Reporting Items for Systematic Reviews and Meta-Analyses (PRISMA) standards based on the Population, Concept, and Context (PCC) framework [15]. Specifically, the Preferred Reporting Items for Systematic Reviews and Meta-Analyses for Scoping Reviews (PRISMA-ScR) checklist was followed. The full protocol has been published [14] and is available on Open Science Framework (OSF) with registration DOI: https://doi.org/10.17605/OSF.IO/7JRWD.

### 2.2. Data Sources and Search Strategy

To identify relevant studies for inclusion, the electronic databases Medline and Embase via Ovid were searched. The search was limited to the period 1 January 2020 to 21 February 2023. The Embase preprints via Ovid search was limited to January 2021 to 21 February 2023. This timeframe for preprints was chosen since it is likely that any papers older than two years would have undergone peer review and been published and, therefore, captured in the main search.

To optimise the search matrix and terms, freely accessible tools such as SR-Accelerator and Polyglot Search Translator [16], Citationchaser [17] and Yale Mesh Analyser [18] were used. Keywords and thesaurus terms were identified, and a pilot study was completed to refine the search strategy and test the literature review management software Covidence [19]. An experienced librarian and content experts were consulted on the search strategy.

#### 2.2.1. Population Concept and Context (PCC) Framework

For the scoping review, a PCC framework was used to develop the search strategy matrix [14] (Supplementary File S1) and was as follows:

##### Population

Papers with a focus on the general adult population and population subgroups that are at an elevated risk of CVD were considered. The elevated risk of CVD subgroup included populations who smoke, consume alcohol, and have a family history of CVD, diabetics, hypertension, cholesterol/dyslipidaemia, obesity, and kidney failure/chronic kidney disease [7,11]. Some papers classified individuals as ‘at risk’ or ‘high-risk’ of CVD due to the presence of clinical features on the pathophysiological pathway to CVD, which included atherosclerosis. Additional groupings in the literature included high CVD risk, those with a history of CVD, revascularisation, or major cardiovascular events. For this review, these populations were grouped and categorised as a ‘high-risk CVD’ population.

##### Concept

*Biomarkers*: For this review, a biomarker is defined as a substance, structure, characteristic, or process that can be objectively measured as an indicator of normal biological processes, pathogenic processes, or biological responses to an exposure [20,21]. Biomarkers were classified into molecular, cellular, imaging, physiological, and anthropometric measures. Each biomarker class contained several subcategories, which are detailed in Table 1. This scoping review maps the current research landscape for novel biomarkers, rather than analysing biomarkers already established in routine clinical practice.

*Diseases*: The cardiovascular diseases included in this scoping review are the main causes of CVD deaths globally and follow the global burden of disease categorisation: ischemic heart disease (IHD), stroke, cardiomyopathy and myocarditis, atrial fibrillation and atrial flutter (AF), aortic aneurysm, nonrheumatic valvular heart disease (NRVHD), and peripheral artery disease (PAD) [7]. Alternate names and subcategories of these disease groups were included. Rheumatic heart disease and endocarditis have not been considered because of their infectious aetiology. Another not considered is heart failure. Although heart failure is a significant burden in healthcare, it is not categorised in the global burden of disease, as it is rather a syndrome that is a consequence of other cardiovascular issues. Therefore, primary and secondary prevention of a CVD should prevent heart failure.

A composite endpoint of diseases was often classified under major adverse cardiac events (MACEs), major adverse cardiac and cerebrovascular events (MACCEs), or cardiovascular events (CVEs) and frequently included CVD-related deaths. Definitions of these terms vary between researchers, and for this review, the term MACE has been used to cover all these categories. Papers that included MACEs were only included when a CVD of interest was named, and the paper met the review criteria. The MACE disease group was challenging to capture, as individual disease breakdown was not always available. Therefore, a MACE category was included in the data extraction form. If a disease breakdown for MACEs was available, both the specific disease and MACE were captured.

*Prevention*: This scoping review focused on personalised primary and secondary prevention for CVD. Papers aimed at preventing or delaying the development of the disease were categorised as primary prevention, while those aimed at identifying patients in the early stages of the disease were categorised as secondary prevention. If the prevention pathway for the biomarker was not specified in the paper, primary or secondary prevention was determined based on the patient’s clinical status (no observable vs. observable clinical manifestations) or the biomarker’s purpose (risk assessment or early detection/ screening), respectively.

For the purposes of this scoping review, papers needed to include the potential use of the biomarker for personalised prevention. In the literature, there is ambiguity and variability in the definition of personalisation [22,23]. As such, if not specified in the paper, we used a broad definition of personalised prevention to cover the research landscape of both primary and secondary prevention. Where biomarkers were assessed as part of a model that would subgroup variables or stratified analyses, these were included in the review. A paper was considered to cover personalised prevention if it attempted to define different disease risk levels and grouped individuals based on the biomarker.

This combination of definitions for personalised prevention may have overestimated the number of biomarkers through the inclusion of papers but avoided the loss of potentially useful papers.

##### Context

This review included papers evaluated within a clinical or public health setting, conducted in any geographical location, and published between January 2020 and mid-February 2023. Only papers published in English were considered.

### 2.3. Eligibility Criteria

This review included papers evaluated within a clinical or public health setting, conducted in any geographical location, and published between January 2020 and mid-February 2023. Studies involving laboratory research in animals, human tissues, and cell lines were excluded. Only papers published in English were considered.

*Inclusion criteria*: Umbrella reviews, systematic reviews, meta-analyses with systematic reviews, scoping reviews, randomised controlled trials, non-randomised controlled trials, cohort studies, case-control studies, and analytical/descriptive cross-sectional studies. English language publications.

*Exclusion criteria*: Editorials, narrative reviews, protocols, qualitative Delphi studies, case reports, guidelines, and conference abstracts. The purpose of this scoping review is the identification of novel biomarkers, and, therefore, well-established CVD biomarkers were excluded (details in Supplementary File S2). These biomarkers were only included when analysed in combination with novel biomarkers. Known monogenic disorders with increased risk for CVD were excluded. Papers that sought to classify patients with the disease according to their possible prognosis (tertiary prevention) were outside the scope of this review. More details are presented in Supplementary File S2.

### 2.4. Screening Process

The screening criteria were established a priori and calibrated through a series of pilot tests [14]. Screening started with dual review and after >90% agreement was observed (achieved when 28% of papers were reviewed). Search results were then screened independently, and discrepancies or challenging papers were resolved through discussion. All screening and data extraction were completed using Covidence.

### 2.5. Data Extraction

A data extraction form (Supplementary File S3) was developed and calibrated using 10% of a random sample of studies.

The Covidence data extraction form captured citation details, study design, disease group, biomarker information, technology used, disease type, prevention type, and at-risk population groups. For primary prevention papers, it included risk factors like smoking, exercise, diet, alcohol consumption, air pollution, obesity, family history, and preventive drug use. Information on technologies, including artificial intelligence (AI), was also captured (Supplementary File S3). Data extraction was completed by two reviewers, with discrepancies resolved through discussion based on the defined definitions and criteria established for primary and secondary prevention, as well as biomarker classification and population groups.

### 2.6. Data Analysis

Results of the data extraction process were exported from Covidence and analysed using the R programming language [24]. Tables summarising the data were created using the R package ‘reactable (v0.4.4)’ [25], and bubble plots were created using the R package ‘ggplot2 (v3.4.4)’ [14]. The R codes are accessible via https://github.com/phg-foundation/PROPHET (accessed on 18 May 2023). Interactive mosaic plots or evidence gap maps (EGMs) [4] available at http://hdl.handle.net/20.500.12105/19630 (accessed on 10 July 2023) were created using Python Version 3 [26] and the EPPI-Mapper tool Version 2.1.0. [27] (Supplementary File S4). It should be noted that most research papers discuss multiple diseases or biomarkers. Therefore, one paper does not necessarily correspond to one disease or one biomarker.

## 3. Results

**Figure 1:**
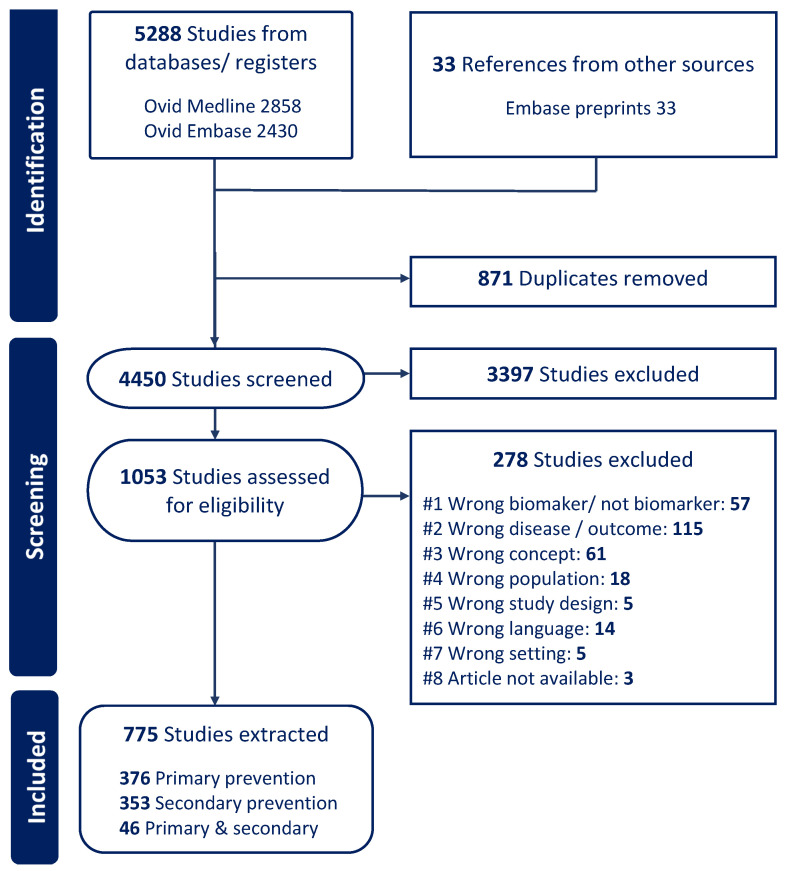
Cardiovascular diseases scoping review PRISMA flowchart.

From the search, a total of 5321 papers were identified, of which 871 were duplicates based on title, author, and year of publication. Of the 4450 papers screened for suitability based on their title and abstract, 1053 papers were retained for full-text review. A further 278 papers were excluded based on full-text review, leaving 775 papers for this review. Ultimately, 376 focused on primary prevention, 353 on secondary prevention, and 46 on both (Figure 1).

### 3.1. Biomarker Research Landscape

Of the CVDs included, the majority (77%, 597/775) of papers investigated IHD (50%, 391/775) and stroke (44%, 338/775), followed by AF (11%, 83/775 papers), across primary and secondary prevention (Table 1). This was consistent across all biomarker groups; therefore, unless otherwise mentioned, the most commonly investigated diseases will be IHD and stroke, followed by AF. The molecular (74%, 575/775) and imaging (23%, 179/775) biomarker categories were the most frequently investigated. For molecular biomarkers, the genetics subcategory (56%, 238/422) was the most commonly investigated in primary prevention and second for secondary prevention of CVD, after the biochemical subcategory (Table 1).

Within the genetics subcategory, single nucleotide polymorphism (SNP) genotyping association studies were the most common study type identified. These studies aim to identify genetic variants associated with disease that could be used to predict disease risk in individuals. These studies investigated single or multiple variants in a single gene or variants across multiple genes. The majority of genes were unique to the papers found, with a small number (*n* ≤ 10) repeated across the papers in the review. The genes that appeared in multiple papers included the methylenetetrahydrofolate reductase (MTHFR) gene and the matrix metalloproteinase-9 (MMP-9) gene. A small number of papers focused on long non-coding RNAs (lncRNAs) or mitochondrial DNA (mtDNA). There were six papers that reported whole-exome or genome-wide association studies (GWAS), which provide broad SNP coverage of the entire genome. The genetic biomarker subcategory included papers looking at Mendelian randomisation (MR) studies. MR uses genetic markers as surrogate measures of features (biochemical biomarkers, protein levels, clinical features such as obesity, hand grip strength, height, lifestyle factors such as smoking, or sleeping habits) to investigate possible causal links with disease [28].

The most investigated molecular subcategory in secondary prevention of CVD was biochemical (40%, 159/399), and this was the second most common molecular subcategory for primary prevention (Table 1). Many biochemical markers were serum markers detectable by standard clinical blood sampling tests, such as high-sensitivity C-reactive protein (hs-CRP), cortisol levels, N-terminal brain natriuretic pro-peptide (NT-proBNP), lipid panels, or uric acid. Refinement in the measurements, measuring derivatives, calculations of ratios, combinations with other clinical features, or biomarkers, as well as their potential use in predicting other diseases, were investigated.

For imaging biomarkers in primary prevention, 13% (56/422) of papers investigated the subcategories of ultrasound (5%, 22 papers), CT scan (3%, 14), and MRI (3%, 12). In 134 imaging biomarker papers investigating secondary prevention, the most common subcategories were MRI (11%, 44/399), CT scan (11%, 45/399), ultrasound (10%, 39/399), and PET/SPECT (4%, 16/399) (Table 1). We identified that, for the early detection of stroke, there was research activity with the use of MRI followed by CT scans.

The physiological, anthropometric, and cellular biomarker groups were the least frequently researched (Table 1). Physiological biomarkers had some activity in primary prevention (9%, 38/422), with more activity in secondary prevention (15%, 60/399), particularly with the use of ECG. This was followed by anthropometric biomarkers (primary 6%, 24/422; secondary 1%, 5/399). Across both primary and secondary prevention, there was limited research being completed on cellular biomarkers (primary 1%, 3/422, secondary 2%, 7/399).

The ‘other’ biomarker category presents findings not captured in the listed categories (Table 1). These included the novel use of retinal-based imaging biomarkers [29,30,31,32,33,34,35] for secondary prevention of CVD and a mammography-based study for IHD [36]. Flow cytometry-based papers were also identified, which evaluated cell count data [37,38,39]. Several new detection methods for physiological biomarkers are also being researched, for example, algorithms that evaluate a range of physical and physiological parameters in the context of CVD [40,41].

Novel technological modalities for biomarker identification (infrared cameras, innovative mass spectrometry techniques, and wearable monitors) that could improve CVD prediction and early detection were found. These included portable imaging devices, which are used to assist ambulance personnel during emergency assessment of risk of stroke outside of the hospital setting [42]. An implantable loop recorder to monitor heart rhythm [43], a seven-day ECG monitor [44,45], and a wrist-worn accelerometer [46] were being used for prediction and early detection of AF. One paper used infrared cameras for early detection of stroke [42]. The use of innovative mass spectrometry technologies [47,48,49,50,51,52,53] and a range of wearable monitors to track cardiac and other physiological parameters were also found [40,44,45,54].

It was observed that some biomarkers used for one specific CVD were being repurposed and evaluated for use in other CVDs. For example, the physiological measurement of the ankle–brachial index is commonly used in PAD, but we found research activity for this biomarker in other CVDs, such as IHD, stroke, and MACE, that included a composite of PAD, IHD, and stroke. Some biomarker subtypes were limited to specific populations; for example, imaging from scintigraphy (gamma) was only being investigated for the risk of IHD in a diabetic population [55].

### 3.2. Interaction with Risk Factors

**Figure 2:**
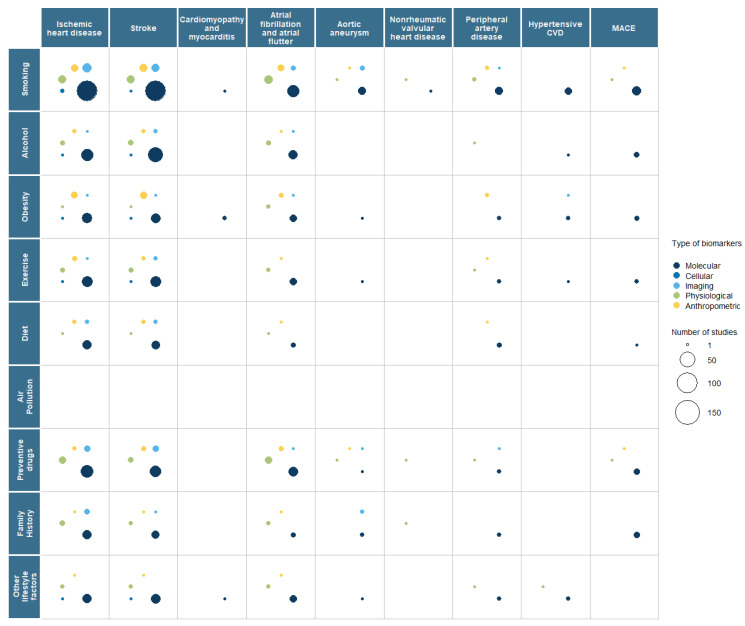
Bubble plot representing the number of papers identified for primary prevention of cardiovascular disease where risk factors were considered; the size of the bubble represents the number of papers, and the colour represents a type of biomarker. CVD—cardiovascular disease; MACE—major adverse cardiovascular event.

For primary prevention of CVD, we captured which risk factors (such as lifestyle or environmental exposures) were being considered if they were a component of the biomarker research, including smoking, exercise, diet, alcohol consumption, air pollution, obesity, family history, and use of preventive drugs. In papers being reviewed, these risk factors were being used as a variable or for adjustment as part of the biomarker analysis. Across commonly investigated disease groups (IHD, stroke, and AF), all risk factors (except air pollution) were taken into consideration (Figure 2). Smoking behaviour was the only risk factor considered across all disease groups. The next most commonly considered risk factors were obesity, exercise, use of preventive drugs, family history, and alcohol consumption. There was limited consideration of diet. Of note was that air pollution, a well-known risk factor for CVD, was not considered in the 775 papers reviewed.

During the review, considerable variability in how these risk factors were considered in the papers was observed, for example, their use as a variable in a regression model, as an adjustment criterion to display demographic data, or in a complex statistical algorithm. Therefore, there is a caveat to the interpretation of the risk factor information, as no inference on causality or association can be made using the data from this scoping review. What can be concluded is that these risk factors are being considered in combination with biomarkers in the research identified.

### 3.3. Role of Artificial Intelligence Technologies

AI technology use was identified for primary and secondary prevention in almost 15% of the 775 papers, more frequently in secondary prevention (Table 2), and usually in conjunction with molecular and imaging biomarkers (Table 2). Novel AI methods that enhance well-established technologies such as CT scanning, ECG, MRI, and ultrasound using machine learning or convolutional neural networks are being researched for their accuracy and utility. For secondary prevention, a fifth of all imaging papers used AI-based methods to aid imaging biomarker research (Table 2).

**Figure 3:**
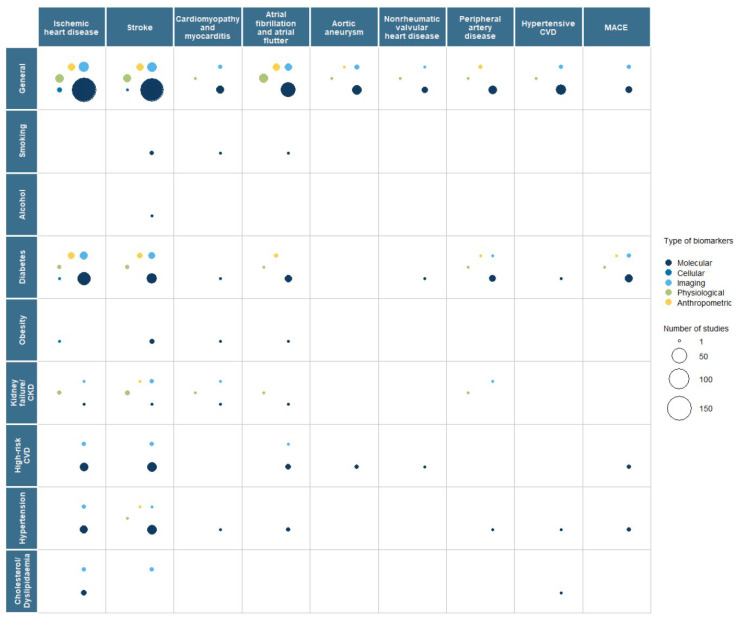
Bubble plot representing the number of papers identified for primary prevention of cardiovascular disease by each population group, where the size of the bubble represents the number of papers, and the colour represents a type of biomarker. No papers looked at family history as a population group in primary prevention. Evidence gap map 1: an interactive version linking to the references identified and used in this bubble plot is accessible via: https://phg-foundation.github.io/PROPHET/Phase_1_codes/EGMs/EGM_cvd_primary_segment_biomarker.html (accessed on 10 July 2023).

**Figure 4:**
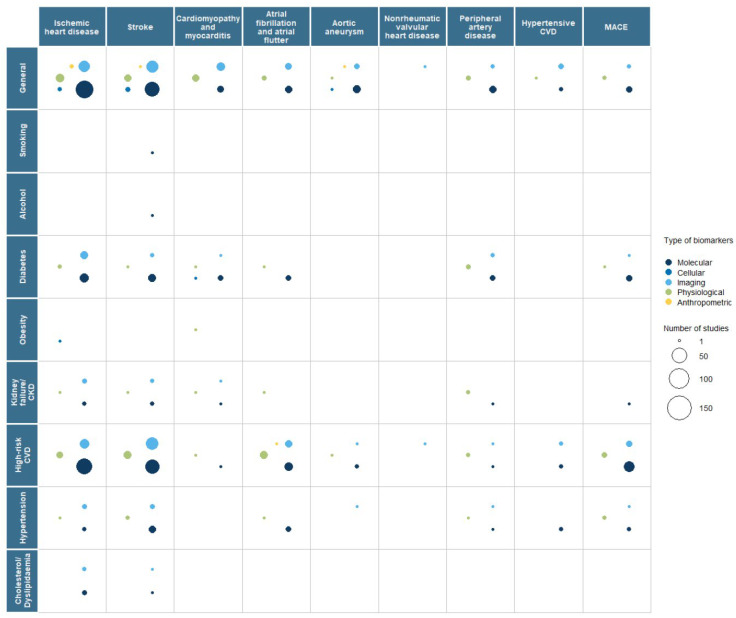
Bubble plot representing the number of papers identified for secondary prevention of cardiovascular disease for each population type, where the size of the bubble represents the number of papers, and the colour represents a type of biomarker. CKD—chronic kidney disease; CVD—cardiovascular disease; MACE—major adverse cardiovascular event. There was one paper that looked at family history as a population group in secondary prevention of cardiomyopathy and myocarditis. Evidence gap map 2: an interactive version linking to the references identified in the scoping review and used in this bubble plot is accessible via: https://phg-foundation.github.io/PROPHET/Phase_1_codes/EGMs/EGM_cvd_secondary_segment_biomarker.html (accessed on 10 July 2023).

Our results show that biomarker research activity in population groups with an elevated risk of CVD was focused on diabetes mellitus in primary prevention and high-risk CVD in secondary prevention (Figure 3 and Figure 4). There was little activity in CKD/kidney failure patients and high cholesterol/dyslipidaemia patients. Limited research was identified in population groups with an elevated risk of CVD based on family history, obesity, smoking, and alcohol consumption (Figure 3 and Figure 4).

## 4. Discussion

In this paper, we present the results of a scoping review of research activity on novel biomarkers for personalised prevention of CVD.

### 4.1. CVD Research Activity and Gaps

The most commonly investigated diseases were IHD and stroke (Table 1), which are the leading causes of CVD death, followed by AF. There was limited or no research activity identified for a number of diseases, e.g., NRVHD, cardiomyopathy, and myocarditis. However, the total number of biomarkers per CVD identified does not correlate with CVD prevalence in the general population. The limited research activity for less prevalent but highly morbid conditions like aortic aneurysm highlights a critical research gap, suggesting an opportunity for targeted investigation rather than a confirmed lack of biomarker potential.

### 4.2. Genetic Biomarkers

Genetic biomarker research has advanced significantly in recent years, and this progress is reflected in our results. Insights into rare Mendelian diseases that aid diagnosis mean genomic medicine has made remarkable progress, offering tailored solutions for patients and their families. It is anticipated that genetic testing will contribute towards personalised prevention of disease. Advances in DNA sequencing enable precision diagnosis, allowing early detection of diseases even before symptoms appear. In the context of CVD, genetic markers could help in assessing risk, guiding lifestyle changes, prioritising screening, and monitoring at-risk patients.

The two main methods used for genetic biomarkers in our review were SNP analysis and PGS. SNP analysis aims to identify associations with disease by testing differences in allele frequencies of genetic variants. However, many of these associations have been weak. To utilise this information, there has been a move towards combining these genetic associations using PGS models. These models combine the information of several to millions of genomic variants, accounting for effect sizes and the aggregated impact of multiple variants. PGS can be developed for the disease of interest (e.g., CAD), clinical features (e.g., hypertension), or a biomarker (e.g., cholesterol or vitamin D levels). In the context of this scoping review, the proposed utility from such genetic information is the possibility of using personalised prevention strategies based on the assessed genetic risk. Genetic biomarkers can be measured in young, asymptomatic adults and present opportunities for the implementation of preventive strategies earlier in the life course.  

### 4.3. Biochemical, Imaging, and Physiological Markers

Biochemical, imaging, and physiological markers often rely on detectable changes in specific molecules or structures, which usually occur during the progression of a disease, even at the sub-clinical level. Biomarkers from these fields have always played a crucial role in healthcare, being incorporated into essential tools for disease detection, monitoring, and personalised treatment. Many biochemical, imaging, and physiological technologies are already commonly available in clinical settings and used for the assessment of patients for the disease of interest, such as ECGs and ankle–brachial measures, CT scans, and X-rays. However, our search of the recent literature identified limited research activity focused on certain established physiological measures, such as pulse wave velocity or flow-mediated dilation, as markers for early vascular ageing, suggesting these may be areas of less intensive current investigation for personalised prevention in the examined period.

In this scoping review, the research was focused on innovative measurement techniques and analyses, including the integration of clinical data with biomarker measurements, in at-risk populations. They also explore the repurposing of these biomarkers for CVDs in addition to those for which they were originally intended. Within our results, AI technologies are being used to assess complex regression models with high volumes of genomic and biochemical data or elaborate imaging files (radiomics). In addition, machine learning algorithms are being used to screen detailed health records. AI, including machine learning algorithms, has a pivotal role in facilitating automated assessment of data generated by these technologies, for example, the use of AI with ECG measurements to provide a more accurate coronary artery calcium (CAC) score for risk prediction of CVD in populations such as those with type-2 diabetes [56,57] or CKD [58]. AI can also be used to review CT scans to detect and triage potential stroke patients in emergency rooms [59,60,61].

### 4.4. Risk Prediction Models

There is growing recognition that combining risk factors with biomarker information enhances prevention strategies by enabling more robust disease prediction. Future applications of biomarkers in prevention will likely involve integrating them into risk scores that use a range of biomarkers from different sources. This scoping review identified such combinations of biomarkers and risk factors and integrated models, which included a broad range of variables [62,63,64,65,66,67,68]. Some studies explored multiple biomarker categories simultaneously, such as multi-omic approaches, to capture various disease-associated modalities and improve risk prediction of a number of diseases [33,69,70]. These combinations aim to facilitate personalised prevention by integrating socio-demographic, lifestyle, and behavioural information with biological data. Assessment of such combinations of variables was outside the scope of this review, but future research will need to consider how to capture this information to inform biomarker development.

### 4.5. Integration with AI

AI was identified in this scoping review, where advances in AI are being used to determine how it can enhance the predictive capabilities of complex, multimodal biomarker data. Risk model research plays a pivotal role in this context, as it helps integrate various biomarkers and other risk factors into comprehensive models that can predict an individual’s risk of developing CVD. AI models allow for the integration of sometimes complex data types to accurately stratify patients based on their calculated risk levels, thereby enabling targeted preventive measures and personalised treatment plans.

### 4.6. Limitations

A known caveat of the scoping review methodology is that it does not assess the methodological quality or potential biases of the papers included. This scoping review focused on identifying research on biomarkers for the prevention of CVD. Scoping reviews are used to identify research priorities and to guide the expansion of research and the generation of new knowledge [71]. Evaluating the clinical utility and integration of these biomarkers into healthcare pathways requires further assessment to fully understand their potential impact and application in clinical settings. As this review synthesises research activity from a broad range of studies, we acknowledge that this may introduce limitations regarding the direct clinical applicability and generalisability of the findings across different populations.

During the title and abstract screening process, a standard and necessary component of scoping review methodology, a large number of papers were excluded. We acknowledge that abstracts may not always fully capture the relevance of a study, and some papers discussing biomarkers in the full text might have been missed if not mentioned prominently. While our broad search strategy was designed to mitigate this, this limitation is inherent to the review process but is unlikely to have materially affected the overall conclusions, given the large volume of literature included.

In this scoping review, we observed inconsistent naming of biomarkers, definitions of disease versus risk factors, and a lack of description regarding the intended prevention pathway or the clinical care pathway where a biomarker would be used. There was variation in how the clinical manifestations of CVDs were described in the papers with a lack of consensus regarding when CVD transitions from sub-clinical (asymptomatic) to symptomatic and which elements were considered disease versus risk factors for CVD (e.g., hypertension and atherosclerosis). The criteria for this scoping review were carefully formulated and piloted to account for this observed heterogeneity in the literature. We also noted that International Classification of Diseases (ICD) coding was rarely used in the reviewed studies, further complicating standardisation. This highlights the critical need for adopting more systematic terminology and classification frameworks in future biomarker research.

Due to the variation and lack of standardisation across the papers for biomarker names (including combinations of biomarkers and their derivatives), the creation of a single biomarker list and a count of the biomarkers reported resulted in more names than papers identified. This demonstrates the need for improved standardisation in the nomenclature of biomarker names.

Apart from the general population, population groups known to be at an elevated risk of CVD were also considered in this scoping review. Research that is specifically focused on populations with an elevated or at high risk of CVD would allow for further personalisation and stratification of risk categories. We acknowledge that our definition of high-risk populations focused on established metabolic and lifestyle risk factors; specific comorbid populations with high CVD risk, such as cancer survivors or patients with chronic inflammatory diseases like rheumatoid arthritis, have distinct pathophysiological pathways and were outside the scope of this review, representing important areas for future work. There are additional study populations that were regularly encountered by the reviewers during the scoping review, namely, ethnicity/country of origin, age, and sex. Furthermore, congenital heart diseases and studies focused on SARS-CoV-2 were explicitly excluded to maintain a clear focus on the prevention of chronic, non-communicable CVD in adults. Similarly, our definition of a biomarker was strictly focused on biological, physiological, or imaging-based measures and did not extend to psychosocial or behavioural risk factors such as chronic stress or sleep quality, which are also known to significantly influence cardiovascular health. Future reviews of the research landscape should take these into consideration.

The reviewers also noted that a core number of well-defined large cohorts and databases, such as the UK Biobank [72], Gene Expression Omnibus (GEO) [73], and MEGASTROKE [74], are being used for the identification of biomarkers. These cohorts and databases house extensive data, allowing for complex analyses without the need for new data gathering. However, relying solely on these resources may perpetuate biases if they do not represent the full spectrum of populations, including demographics and ethnic diversity, and can have an impact on the wider utility of the application of biomarkers developed from these. It is crucial to ensure diverse representation in biomarker research to avoid potential biases and obtain more comprehensive, accurate, and widely applicable results. Understanding which cohorts are being utilised for research could also provide a better understanding of the limitations of the research landscape for biomarkers.

Despite significant improvements over the last few decades in decreasing air pollution, this exposure remains the largest environmental health risk [75,76]. Its absence from the papers may be due to the complexity in capturing individual exposure to air pollution.

## 5. Conclusions

This scoping review provides details regarding the extensive research activity on biomarkers for the personalised prevention of CVD. Most studies identified focused on IHD and stroke, reflecting their prevalence and impact on public health. Significant research activity for early detection and personalised prevention strategies is taking place with genetic biomarkers, particularly SNP analysis and polygenic scores.

Despite this progress, there are notable gaps in research, particularly for less common CVDs such as aortic aneurysm and nonrheumatic valvular heart disease. Additionally, the variability in how CVD is classified, the lack of clarity on the use of specific biomarkers in prevention pathways, and the limited consideration of certain risk factors, such as air pollution, highlight areas for future research. The integration of AI is emerging as a key enabler, helping to analyse complex, multimodal data to enhance the predictive power of these biomarkers. Ensuring diverse representation in biomarker research and integrating biomarkers with risk factors will be essential for developing comprehensive and effective personalised prevention strategies.

## Figures and Tables

**Table 1:** Number of papers found for primary and secondary prevention by biomarker categories and cardiovascular disease.

	Primary Prevention										Secondary Prevention									
Biomarker	Total	IHD	Stroke	Cardiomyopathy Myocarditis	AF	AA	NRV HD	PAD	Hypertensive CVD	MACE	Total	IHD	Stroke	Cardiomyopathy Myocarditis	AF	AA	NRV HD	PAD	Hypertensive CVD	MACE
**Total**	**422**	**227**	**198**	**11**	**67**	**19**	**7**	**20**	**21**	**21**	**399**	**191**	**157**	**24**	**43**	**20**	**2**	**22**	**12**	**45**
***Molecular***	361	190	172	10	55	17	6	17	20	17	247	135	96	10	22	12	0	13	6	33
Genetics	238	120	117	9	38	35	6	9	16	7	39	20	14	2	2	3	0	2	0	3
Epigenetics	7	3	6	0	0	0	0	0	0	0	4	1	2	1	0	0	0	0	0	0
Transcriptomics	26	9	13	0	3	1	1	0	2	0	31	14	10	2	4	3	0	0	1	1
Metabolomics	8	4	5	0	1	0	0	1	1	0	18	11	9	2	0	1	0	0	1	2
Proteomics	10	5	3	0	2	1	1	0	0	0	23	11	7	1	1	2	0	3	1	1
Microbiomics	1	1	0	0	1	0	0	0	1	0	1	1	0	0	0	0	0	0	0	0
Biochemical	119	72	52	1	17	4	0	8	4	11	163	88	69	5	18	7	0	8	4	27
Other	1	1	0	0	0	0	0	0	0	0	0	0	0	0	0	0	0	0	0	0
***Cellular***	3	3	1	0	0	0	0	0	0	0	7	2	3	1	0	1	0	0	0	0
Histology	0	0	0	0	0	0	0	0	0	0	1	0	0	0	0	1	0	0	0	0
Cytology	1	1	0	0	0	0	0	0	0	0	1	1	0	0	0	0	0	0	0	0
Other	2	2	1	0	0	0	0	0	0	0	5	1	3	1	0	0	0	0	0	0
***Imaging***	56	31	24	3	8	3	1	2	2	4	134	48	60	13	13	6	2	6	6	9
X rays	2	1	0	1	0	0	0	0	0	0	7	3	1	1	0	0	1	2	0	1
Ultrasound	22	8	8	2	5	1	0	2	1	1	39	10	12	6	5	4	1	3	1	1
CT scan	14	13	5	0	1	0	0	0	0	1	45	18	24	1	5	3	0	0	1	4
PET/ SPECT	5	4	1	0	0	0	0	0	0	1	16	11	2	2	1	1	0	0	0	1
Spectrometry	0	0	0	0	0	0	0	0	0	0	2	0	1	1	0	0	0	0	0	0
MRI	12	1	6	3	3	1	1	0	1	0	44	6	29	7	6	1	0	1	4	2
Scintigraphy (Gamma)	0	0	0	0	0	0	0	0	0	0	0	2	0	0	0	0	0	0	0	0
Mammography	0	0	0	0	0	0	0	0	0	0	1	1	0	0	0	0	0	0	0	0
Other	7	5	4	0	1	1	0	0	0	1	0	2	2	0	0	0	0	1	0	0
***Physiology***	38	15	14	2	15	1	1	3	1	1	60	21	19	10	13	2	0	7	1	8
Blood pressure	16	6	7	0	7	1	0	1	0	1	16	4	7	2	2	1	0	1	0	2
Ankle-brachial index	2	1	1	0	0	0	0	1	0	0	4	1	1	0	0	0	0	0	0	1
ECG	14	3	2	2	9	0	1	0	0	0	30	10	4	8	11	1	0	0	1	2
EEG	1	0	1	0	0	0	0	0	0		1	0	1	0	0	0	0	0	0	0
Electromyography	1	0	1	0	0	0	0	0	0	0	0	0	0	0	0	0	0	0	0	0
Other	11	5	5	0	2	0	0	2	1	0	11	9	8	2	0	1	0	4	0	4
***Anthropometric***	24	12	11	0	8	1	0	2	0	1	5	2	1	0	1	1	0	0	0	0
BMI	13	7	6	0	5	0	0	1	0	1	3	2	1	0	0	0	0	0	0	0
Body perimeters	8	5	4	0	2	0	0	1	0	0	0	0	0	0	0	0	0	0	0	0
Other	9	4	4	0	2	1	0	0	0	1	1	0	0	0	1	1	0	0	0	0

AF—atrial fibrillation and atrial flutter; AA—aortic aneurysm; IHD—ischemic heart disease; NRVHD—nonrheumatic valvular heart disease; PAD—peripheral artery disease; MACE—major acute cardiac event; CT—computed tomography; PET—positron emission tomography; SPECT—single-photon emission computerized tomography; MRI—magnetic resonance imaging; ECG—electrocardiogram; EEG—electroencephalogram; BMI—body mass index. Numbers cannot be added up to give totals, as some papers would look at more than one CVD or more than one type of biomarker.

**Table 2:** Number of papers found using artificial intelligence for primary and secondary prevention by biomarker categories for cardiovascular diseases.

	Total	Ischemic Heart Disease	Stroke	Cardiomyopathy and Myocarditis	AF	AA	NVHD	PAD	Hypertensive CVD	MACE
Primary prevention										
**Total**	**22**	**10**	**9**	1	8	2	0	**0**	**0**	**0**
Molecular	**15**	6	4	1	6	2	0	0	0	0
Cellular	0	0	0	0	0	0	0	0	0	0
Imaging	**9**	4	4	1	4	1	0	0	0	0
Physiological	**7**	1	3	0	4	0	0	0	0	0
Anthropometric	**1**	0	1	0	0	0	0	0	0	0
**Secondary prevention**										
**Total**	56	24	18	4	8	4	1	3	2	1
Molecular	**22**	15	7	1	1	2	0	**1**	0	0
Cellular	0	0	0	0	0	0	0	0	0	0
Imaging	**28**	8	11	2	3	**2**	1	**1**	2	**1**
Physiological	**15**	4	5	1	5	0	0	**1**	0	0
Anthropometric	**1**	1	0	0	0	0	0	0	0	0

## Data Availability

Tables summarising the data were created using R package ‘reactable (v0.4.4)’, and bubble plots were created using R package ‘ggplot2 (v3.4.4)’. The R codes are accessible via https://github.com/phg-foundation/PROPHET (accessed on 18 May 2023). Interactive mosaic plots or evidence gap maps (EGMs) [4] are available at http://hdl.handle.net/20.500.12105/19630 (accessed on 12 April 2023).

## Supplementary Materials

The following supporting information can be downloaded at: https://www.mdpi.com/article/10.3390/ijms26199346/s1.

## Abbreviations

The following abbreviations are used in this manuscript:

AA	Aortic Aneurysm
AF	Atrial Fibrillation and Atrial Flutter
AI	Artificial Intelligence
BMI	Body Mass Index
CAD	Coronary Artery Disease
CAC	Coronary Artery Calcium
CKD	Chronic Kidney Disease
CT	Computed Tomography
CVE	Cardiovascular Event
CVD	Cardiovascular Disease
ECG	Electrocardiogram
EEG	Electroencephalogram
EGMs	Evidence Gap Maps
EU	European Union
GEO	Gene Expression Omnibus
GWAS	Genome-Wide Association Study
hs-CRP	High-Sensitivity C-Reactive Protein
IARC	International Agency for Research on Cancer
IHD	Ischemic Heart Disease
lncRNA	Long Non-Coding RNA
MACCE	Major Adverse Cardiac and Cerebrovascular Event
MACE	Major Acute Cardiac Event
MEGASTROKE	Multiancestry Genome-Wide Association Study
MRI	Magnetic Resonance Imaging
MR	Mendelian Randomisation
mtDNA	Mitochondrial DNA
NCD	Non-Communicable Disease
NICE	National Institute for Health and Care Excellence
NRVHD	Nonrheumatic Valvular Heart Disease
NT-proBNP	N-terminal Brain Natriuretic Pro-Peptide
OSF	Open Science Framework
PAD	Peripheral Artery Disease
PET	Positron Emission Tomography
PCC	Population, Concept, Context
PGS	Polygenic Score
PRISMA-ScR	Preferred Reporting Items for Systematic Reviews and Meta-Analyses for Scoping Reviews
PROPHET	PeRsOnalised Prevention Roadmap for the Future HEalThcare
SNP	Single Nucleotide Polymorphism
SPECT	Single-Photon Emission Computed Tomography
SRIA	Strategic Research and Innovation Agenda
UK	United Kingdom
WHO	World Health Organisation
