# Supplementary material for: Biomarkers for Personalised Primary or Secondary Prevention in Cardiovascular Diseases: A Rapid Scoping Review
Source: Int J Mol Sci. 2025 Sep 24;26(19):9346. doi: 10.3390/ijms26199346 (PMC12524637; doi:10.3390/ijms26199346)
Supplement: Supplementary file 1 [file ijms-26-09346-s001.zip › ijms-3822415-supplementary.pdf]

Review

Biomarkers for personalised primary or secondary prevention in cardiovascular diseases: a rapid scoping review

Supplementary files

Supplementary file 1. Search strategies for the different databases and repositories.

The PCC framework was considered to develop the search strategy, and the following topics were obtained for the search: biomarkers, cardiovascular diseases, prevention and personalised. For each of them, the corresponding indexed and free terms were included. As detailed below, "blocks" (biomarkers, diseases, prevention, and personalised) using the "AND" operator to be combined following this general strategy during the search:

#1 biomarkers
#2 diseases
#3 prevention
#4 personalised, precision, prediction or predictive
#5 #1 AND #2 AND #3 AND #4
#6 #5 Filters: from 2020-2023

Our MeSH and free text keyword terms were identified through a rigorous, systematic, multi-step process. The selection began with expert input from content specialists to define the diseases of interest. We then conducted a formal analysis using specialized tools, such as the tools such as SR-Accelerator & Polyglot Search Translator [16], Citationchaser [17] and Yale Mesh Analyzer [18], for an evidence-based selection of the most appropriate terms. The resulting search strategy was subsequently validated by an experienced medical librarian, and finally, it was refined through a pilot study to ensure its accuracy and effectiveness before the full review was conducted. Despite this rigorous, multi-step process, we acknowledge that any search strategy is inherently limited by the initial scope defined for the review and the dynamic nature of database indexing and scientific terminology.

OVID MEDLINE MESH
**BIOMARKERS**
(exp Biological factors/ or ("biological factor*" or biomarker* or "bio* marker*").ti,ab,kw,kf.)
(exp *carbohydrates/ or exp *lipids/ or exp *amino acids/ or exp *proteins/ or exp *vitamins/ or exp *steroids/ or exp *Hormones/ or exp *Enzymes/)
(exp genetic markers/ or exp genotype/ or exp genetics/ or exp genomics or ("genetic marker*" or "genotype" or "germline biomarker*" or "genetic*" or "genomic*" or "epigen*" or "epigenetic mark*").ti,ab,kw,kf.)
(exp Microchip Analytical Procedures/) or (Microarray*).ti,ab,kw,kf.
(exp Metabolomics/ or exp Proteomics/ or ("metabolomic*" or "proteomic*" or "lipidomic*" or "immunomic*" or "nutriomic*" or "transcriptomic*" or "radiomic*").ti,ab,kw,kf.)
(exp *Diagnostic Imaging/ or imag*.ti,ab,kw,kf.)

DISEASES
(exp *atrial fibrillation/ or exp *atrial flutter/ or 'atrial fibrillation'.ti,ab,kw,kf.)
(exp *cardiomyopathies/ or exp *myocarditis/ or 'cardiomyopath*'.ti,ab,kw,kf. Or 'myocarditis'.ti,ab.)
exp *myocardial ischemia/ or exp *myocardial infarction/ or 'ischemic heart disease'.ti,ab,kw,kf. or 'ischaemic heart disease'.ti,ab,kw,kf. Or 'heart attack'.ti,ab,kw,kf.
exp *heart valve diseases/ OR ("Nonrheumatic valvular heart" OR "calcific aortic valve" OR "degenerative mitral valve" OR "Nonrheumatic heart").ti,ab,kw,kf.
exp *Peripheral Arterial Disease/ or 'Peripheral Arterial Disease'.ti,ab,kw,kf.
exp *Aortic Aneurysm/ OR '"aortic aneurysm".ti,ab,kw,kf.
exp *stroke/ or exp *brain infarction/ or exp *hemorrhagic stroke/ or exp *ischemic stroke/ or 'stroke'.ti,ab,kw,kf. OR exp *Cerebral Hemorrhage/ OR exp *Subarachnoid Hemorrhage/

PREVENTION
(exp Primary Health Care/ or ("public health service*" or "primary health care" or "primary care").ti,ab,kw,kf.)
(exp Primary prevention/ or ("primary disease prevent*" or "preventable disease*").ti,ab,kw,kf.)
(exp Preventive Medicine/ or ("preventive medicine*" or "medical prevent*").ti,ab,kw,kf.)
("protective factor*".ti,ab,kw,kf.)
(susceptibil*.ti,ab,kw,kf.)
(exp *smoking/)
(exp *exercise/ or exp *sedentary behavior/)
(exp *"Diet, food, and nutrition"/ or exp *Overweight/)
(exp *Alcohol Drinking/)
(exp *Type 2 Diabetes Mellitus/)
exp *renal insufficiency, chronic/ or exp *kidney failure, chronic/
(exp *air pollution/)
((exp *blood pressure/ or exp *hypertension/) or ("blood pressure" or "hypertension" or "high blood pressure").ti,ab,kw,kf.)
(exp *hypercholesterolemia/ OR exp lipid metabolism disorders/ or exp dyslipidemias/ or "dyslipedemia*".ti,ab. OR "dyslipidaemia*".ti,ab,kw,kf. OR "high cholesterol".ti,ab,kw,kf.)
exp hyperglycemia/ or exp *Blood Glucose/ or "high blood glucose".ti,ab,kw,kf.
(exp *Community Health Planning/ or ("community setting*" or "health care plan*" or "community health plan*").ti,ab,kw,kf.)
(exp *Preventive health service/ or "preventive health service*".ti,ab,kw,kf.)
(exp *Community Health Center/ or ("community health center*" or "community health centre*" or "community care").ti,ab,kw,kf.)
(exp *Health Education/ or ("health educat*" or "health promot*").ti,ab,kw,kf.)
(exp Mass Screening/ or exp Secondary Prevention/ or ("secondary prevent*" or "screening*").ti,ab,kw,kf.)
(exp Population Surveillance/ or ("population surveillance*" or "population screening*").ti,ab,kw,kf.)
(exp Early Diagnosis/ or ("early diagnos*").ti,ab,kw,kf.)

PERSONALISED
(exp Precision medicine/ or ("personalised medicine*" or "precision medicine*" or "predict*" or "individualized medicine*" or "personalised medicine*" or "individualised medicine*").ti,ab,kw,kf.)
(exp Risk assessment/ or exp Risk adjustment/ or ("risk stratifi*" or "risk scor*" or "risk assess*" or "risk adjust*").ti,ab,kw,kf.)
(exp Machine learning/)

OVID EMBASE EMTREE
BIOMARKERS
(exp marker/ or ('biological factor*' or 'bio* marker*').ti,ab.)
(exp *'carbohydrate'/ or exp *'lipid'/ or exp *'amino acid'/ or exp *'protein'/ or exp *'vitamin'/ or exp *'steroid'/ or exp *'hormone'/ or exp *'enzyme'/)
(exp 'genetic marker'/ or exp 'genotype'/ or exp 'genetics'/ or exp 'genomics'/ or ('genotype' or 'genetic*' or 'genomic*' or 'epigenetic mark*' or 'germline biomarker*' or 'epigen*' or 'genetic marker*').ti,ab.)
(exp 'microchip analysis'/ or exp 'microarray analysis'/ or ('microarray*').ti,ab.)
(exp metabolomics/ or exp proteomics/ or exp lipidomics/ or exp nutriomics/ or exp transcriptomics/ or exp immunomics/ or exp radiomics/ or (proteomic* or lipidomic* or nutriomic* or metabolomic* or transcriptomic* or immunomic* or radiomic*).ti,ab.)
(exp *'Diagnostic Imaging'/ or (imag*).ti,ab.)

DISEASES
exp *atrial fibrillation/ or exp *heart atrium flutter/ or 'atrial fibrillation'.ti,ab.
exp *cardiomyopathy/ or exp *myocarditis/ or 'cardiomyopath*'.ti,ab. or 'myocarditis'.ti,ab.
exp *heart muscle ischemia/ or exp *heart infarction/ or 'ischemic heart disease'.ti,ab. or 'ischaemic heart disease'.ti,ab.
exp *aortic valve disease/ or exp *mitral valve disease/ or "nonrheumatic heart".ti,ab.
exp *peripheral occlusive artery disease/ OR 'Peripheral Arterial Disease'.ti,ab.
exp aortic aneurysm/ OR "aortic aneurysm".ti,ab.
exp *cerebrovascular accident/ or exp *ischemic stroke/ OR exp *brain hemorrhage/ OR exp *subarachnoid hemorrhage/ OR 'Stroke'.ti,ab.

PREVENTION
(exp 'primary health care'/ or exp 'public health service'/ or ('primary health care' or 'primary care' or "public health service*").ti,ab)
(exp 'primary prevention'/ or ('primary disease prevent*' or 'preventable disease*').ti,ab.)
(exp 'preventive medicine'/ or ('medical prevent*' or 'preventive medicine*').ti,ab.)
('protective factor*'.ti,ab.)

(susceptibil*.ti,ab.)
(exp *smoking/)
(exp *exercise/ or exp *'sedentary lifestyle'/)
(exp *nutrition/ or exp *obesity/)
(exp *'drinking behavior'/ or exp *'alcohol consumption'/)
(exp *'non insulin dependent diabetes mellitus'/)
(exp *hypercholesterolemia/ OR exp dyslipidemia/ or "dyslipidemia*".ti,ab. OR "dyslipidaemia*".ti,ab. OR "high cholesterol".ti,ab.)
(exp *'blood pressure'/ or exp *'hypertension'/ or ('blood pressure' or 'high blood pressure' or 'hypertension').ti,ab.)
exp hyperglycemia/ OR exp glucose blood level/ or "high blood glucose".ti,ab.
exp *kidney failure/ or exp *chronic kidney failure/
exp heart infarction prevention/
(exp *'air pollution'/)
('health care plan*' or 'community setting*' or 'community health plan*').ti,ab.
(exp *'preventive health service'/ or ("preventive health service*").ti,ab.)
(exp community care/ or ('community health center*' or 'community health centre*' or 'community care').ti,ab.)
(exp *'health education'/ or exp *'health promotion'/ or ('health educat*' or 'health promot*').ti,ab.)
(exp 'mass screening'/ or exp 'secondary prevention'/ or ('screening*' or 'secondary prevent*').ti,ab.)
(exp 'population surveillance'/ or ('population surveillance*' or 'population screening*').ti,ab.)
(exp Early Diagnosis/ or ("early diagnos*").ti,ab.)

**PERSONALISED**
(exp 'personalized medicine'/ or ('precision medicine*' or 'predict*' or 'personalized medicine*' or 'individualized medicine*' or 'personalised medicine*' or 'individualised medicine*').ti,ab.)
(exp 'risk assessment'/ or ('risk stratif*' or 'risk scor*' or 'risk adjust*' or 'risk assess*').ti,ab.)
(exp 'Machine learning'/)

Supplementary file 2. Eligibility criteria.

**Inclusion**	**Exclusion**
**Population**<br>✓ Adult general population (> 18 years) (this results in the exclusion of congenital heart disease from this scoping review)<br>✓ Apart from the general population, we have included relevant specific subgroups of high-risk adults depending on the group of diseases to which they belong: Smoking, alcohol consumption, diabetes, obesity, family history, hypertension, hypercholesterolemia or dyslipidaemia, APOE genotype, hearing impairment.<br>✓ People who have already had the disease of interest, have it or in whom there is a proxy for the disease.<br>✓ Any country of origin	**Population**<br>× Pregnancy<br>× SARS-CoV2 infections related
**Concept**<br>✓ Studies with a focus on molecular, cellular, physiological, and imaging biomarkers used for individualised primary or secondary<br>prevention of amyotrophic lateral sclerosis, Parkinson's disease, Alzheimer's disease, frontotemporal dementia, vascular dementia, Lewy Body disease and multiple sclerosis.<br>✓ In primary prevention: Biomarkers should stratify individuals into groups<br>✓ In secondary prevention: Biomarkers should improve the screening or early detection:<br>◦ stratifying different risk groups<br>◦ improving the sensitivity, specificity, and time of diagnosis<br>for new subgroups of individuals<br>✓ Studies with some risk stratification related to the early detection<br>(not to the evolution of the disease)	**Concept**<br>× Basic research in animals, human tissues and cell lines
**Context**<br>✓ Clinical or public health settings<br>✓ English language only<br>✓ Any geographical setting	**Context**<br>× Studies published before 2020

**Type of evidence**<br>✓ Reviews:<br>Umbrella review, systematic review, meta-analysis, scoping review<br>✓ Experimental and quasi-experimental study designs:<br>Randomised controlled trials, non-randomised controlled trials, before and after studies, interrupted time-series studies<br>✓ Analytical observational study designs:<br>Prospective and retrospective cohort studies, case–control studies, and analytical cross-sectional studies<br>✓ Descriptive observational study designs:<br>Descriptive cross-sectional studies<br><br>**Sources**<br>✓ MEDLINE via Ovid<br>✓ Embase via Ovid<br>✓ Embase preprints via Ovid	**Type of evidence**<br>× Editorials and opinion pieces<br>× Narrative reviews<br>× Protocols<br>× Qualitative study designs. Delphi studies<br>× Conference abstracts, conference reports<br>× Clinical practice guidelines<br>× Basic research (i.e. laboratory research in animals, human tissues, and cell lines)<br>× Data simulation or modelling studies<br>× Case reports and case series

Biomarkers defining the pre-specified high-risk subpopulations

Condition	Known biomarkers
Diabetes	plasma glucose, fasting plasma glucose, blood sugar levels, glycaemia, glycated haemoglobin, insulin resistance.
Obesity	BMI, weight, waist circumference, waist-hip ratio (WHR) .
Hypertension	(high) blood pressure.
Cholesterol/ dyslipidaemia	high-density lipoprotein (HDL), low-density lipoprotein (LDL), very low-density lipoprotein (VLDL), Intermediate-density lipoproteins (IDL), cholesterol, atherosclerosis, triglycerides
Smoking	Cotinine.
Alcohol consumption	Blood alcohol level, blood/breath alcohol concentration/content (BAC), ethanol level.
Kidney disease	Serum Creatinine (SCr), Glomerular Filtration Rate (GFR), Cystatin C (CysC), Kidney Injury molecule 1(KIM-1), Neutrophil gelatinase-associated lipocalin (NGAL), Liver-type fatty acid–binding protein (L-FABP), Uromodulin.
Exercise	Metabolic equivalents (METs).
Air pollution	Oxidative stress (GDF-15 and myeloperoxidase), acute inflammation (C-reactive protein), hemostasis (ADAMTS, D-dimer)
CVD-specific	Troponin

It should also be noted that complex indices or scores derived from combinations of well-established biomarkers (e.g., Atherogenic Index of Plasma, Castelli Risk Index) and conditions (e.g., metabolic syndrome) were generally considered part of the established risk assessment landscape. They were only included in this review if they were being validated as a novel biomarker in a new predictive context that otherwise met the inclusion criteria

Supplementary file 3. Data extraction form options that were in Covidence.

The data extraction form in Covidence allowed for the capture of citation details, study design (randomised control trial, cohort, case control, review and other), disease group (IHD, cardiomyopathy and myocarditis, AF, aortic aneurysm, NRVHD, PAD, stroke, hypertensive CVD and MACE), biomarker information (name, type, subtype), technology used (AI, smart watch, pulse oximetry, infrared cameras and other, other), disease type (group, specific disease), prevention type (primary or secondary), and at risk population groups (smoking, alcohol, diabetes, obesity, chronic kidney disease/kidney failure, hypertension, family history, high cholesterol/dyslipidaemia and high risk CVD). A free text field was also included to allow reviewers to capture any relevant observations from the papers. For primary prevention papers, the data extraction sheet allowed for capture of risk factors assessed alongside a biomarker such as lifestyle factors and

included: smoking, exercise, diet, alcohol consumption, air pollution, obesity, family history and use of preventive drugs.

Information on the use of various technologies could also be captured. The use of wearable technology included personal sensors or computing devices incorporated into accessories to provide healthcare information about people wearing them. Artificial intelligence was also considered under technologies, and when considering healthcare we included image processing, computer vision, artificial neural network (ANN), machine learning, convolutional neural network (CNN) and deep learning.

The IHD category included coronary artery disease (CAD), myocardial infarction, heart attack, heart muscle ischemia, heart infarction, coronary heart disease, myocardial ischemia and acute coronary syndrome (ACS). The stroke category included ischemic stroke (including embolic stroke and transient ischemic stroke), intracerebral haemorrhage, and subarachnoid haemorrhage.

**Data Extraction form CODE**		
Methods	Study design	Umbrella reviews<br>Systematic review (With meta analysis)<br>Systematic review (Without meta analysis)<br>RCT<br>Cohort study<br>Case-control study<br>Other design<br>N/P (Not Provided)
	Mendelian Randomization	Has Mendelian randomization been used in the study?
Biomarker(s)	Name	Name of the biomarker(s) in text format
	Molecular	Genetics/Genomics<br>Epigenetics/Epigenomics<br>Transcription/Transcriptomics<br>Metabolites/Metabolomics<br>Proteins/Proteomics<br>Microbiomics/Microbiology<br>Biochemistry<br>Other molecular biomarker<br>N/P
	Cellular	Histology (tissue abnormalities)<br>Cytology (cell types)<br>Other cellular biomarker<br>N/P
	Imaging	X-Rays<br>Ultrasound (echography, etc)<br>CT Scan<br>PET/SPECT<br>Spectrometry<br>MRI<br>Scintigraphy (Gamma)<br>Other image biomarker<br>N/P
	Physiological	Blood Pressure<br>Ankle-brachial Index<br>Electrocardiogram<br>Electromiography<br>Other physiological biomarker<br>N/P
	Anthropometric measures	Body mass index<br>Body perimeters<br>Other anthropometric biomarker<br>N/P

**Data Extraction form CODE**		
Clinical Utility	Does the paper mention the clinical utility of the biomarker? (explicitly)	
AI	Did they use AI technology or methods related to AI? (Deep learning, machine learning, clinical trial simulation, etc.)	
Radiomics	Does the paper mention radiomics?	
Technology	If they used a new technology/wearable to measure the biomarker, specify which one	Smart watch<br>Pulse oximetry<br>Infrared cameras<br>Other
Diseases	CVD	Ischemic heart disease<br>Cardiomyopathy and myocarditis<br>Atrial fibrillation and atrial flutter<br>Aortic aneurysm<br>Nonrheumatic valvular heart disease<br>Peripheral artery disease<br>Stroke<br>Stroke -ischemic stroke<br>Stroke - intracerebral haemorrhage<br>Stroke - subarachnoid haemorrhage<br>Hypertensive heart disease<br>MACE
Prevention	Type	Primary<br>Secondary: Screening, early diagnosis, and early detection<br>N/P
	If Primary: Lifestyle (Risk factors)	Smoking<br>Exercise<br>Diet<br>Alcohol<br>Air pollution<br>Obesity<br>Preventive drugs<br>Other: Specify which one<br>N/P
Population	Population type	General: without any other condition or healthy participants. (Included in all disease groups)<br>Smoking<br>Alcohol consumption<br>Family history<br>Diabetes<br>Hypertension<br>Dyslipidaemia<br>Obesity<br>Kidney failure / chronic kidney disease<br>N/P
Observations		

Supplementary file 4. Eligibility criteria. Definitions.

Prevention: reduction of the likelihood of developing a disease, sustaining an injury, or experiencing an unfavourable outcome. This study includes both collective (e.g., population screening) and individual (e.g., individual risk stratification) preventive measures.

Primary prevention is targeted at individuals that have not developed clinical manifestations of the diseases but are considered at risk of developing them, thus efforts are focused on preventing the onset of the disease.

Secondary prevention involves the time in which an intervention might be made to avoid disease progression. Screening programmes also play a role in secondary prevention. Screening is actively offered to defined population groups who do not have symptoms and have not sought medical help but are defined as being at risk of the disease.

Biomarkers were classified in the following categories:

1. **Molecular Biomarkers**: These biomarkers involve the analysis of specific molecules such as DNA, RNA, proteins, metabolites, or genetic variations. This category encompasses both studies using specific, more targeted molecules and those using high-throughput, hypothesis-free approaches such as arrays. They are subclassified into the following groups: genetic/ genomics; epigenetic/ epigenomics; transcriptomics; metabolites/ metabolomics; proteins/ proteomics; microbiological/ microbiomics; biochemistry, and others. Within genetic/genomics, we have also extracted specifically information on the use of mendelian randomization.
2. **Cellular Biomarkers**: In this category, we have included both cytological and histological biomarkers. These include the analysis of cells or cell components to provide information about cellular processes, dysfunction, or disease states, or examination of tissue samples through techniques such as staining or immunohistochemistry to identify specific cellular or tissue characteristics associated with a disease.
3. **Physiological Biomarkers**: Physiological biomarkers reflect functional changes in the body and can include measurements such as blood pressure or heart rate. This group also includes patterns of movement (i.e. derived from accelerometery).
4. **Imaging Biomarkers**: Imaging biomarkers utilize medical imaging techniques, such as X-rays, MRI (magnetic resonance imaging), PET (positron emission tomography), or CT (computed tomography), to visualize and measure structural, functional, or molecular changes in organs or tissues.
5. **Anthropometric Biomarkers**: Anthropometric biomarkers involve the measurement of physical characteristics of the body, such as body mass index (BMI), waist circumference, or body fat percentage. In almost all the cases, they are covariables in multivariate models that include other biomarkers and/or personal characteristics of the individuals.

Lifestyles and subpopulations explored: the strategy we have used to incorporate existing information and knowledge on prevention into the review is as follows:

In primary prevention, there are lifestyle-related factors that, similar to family history, clearly influence the likelihood of developing the disease. Biomarkers should add additional value to what it is already known. In addition, we have explored, both in primary and secondary prevention, whether scientific papers examined differences or provided specific risk estimators for subgroups defined by certain risk factors and/or clinical conditions or diseases associated with clear increases in the risk of our target diseases. These studies are likely to be particularly valuable as they could help refine or personalised prevention strategies.

Wearable technology, the use of personal sensors or computing devices incorporated into accessories to provide healthcare information about people bearing them.

Artificial intelligence, when referring to the medical field, includes mainly image processing, computer vision, artificial neural network (ANN), machine learning, convolutional neural network (CNN) and Deep learning (DL).
